# Excellent hemostatic control during cardiac surgery in a patient with hemophilia B treated with albutrepenonacog alfa (rIX‐FP): A case report

**DOI:** 10.1002/ccr3.7439

**Published:** 2023-06-15

**Authors:** Francisco José López‐Jaime, Ihosvany Fernández‐Bello, Eva Calavia‐Aranda, Begoña Reina‐Monsó, Adrián Montaño

**Affiliations:** ^1^ Unidad de Hemostasia y Trombosis Hospital Universitario Regional de Málaga, IBIMA Malaga Spain; ^2^ Servicio de Cardiología Hospital Universitario Regional de Málaga, IBIMA Malaga Spain; ^3^ Universidad de Salamanca Salamanca Spain

**Keywords:** cardiac surgery, FIX, hemophilia B, rIX‐FP

## Abstract

**Key Clinical Message:**

In hemophilia patients undergoing cardiac surgery, ROTEM® and Quantra® viscoelastic tests are useful to monitor perioperative hemostatic status and a single dose of rIX‐FP is safe and avoids any hemorrhagic or thrombotic complication.

**Abstract:**

Cardiac surgery poses a high hemostatic risk in patients with hemophilia. We present the first case of an adult patient with hemophilia B on treatment with albutrepenonacog alfa (rIX‐FP) who underwent surgery for acute coronary syndrome. Treatment with rIX‐FP made it possible to perform the surgery safely.

## 1 INTRODUCTION

Hemophilia is an inherited bleeding disorder caused by deficiency of clotting factors VIII (FVIII) in hemophilia A and factor IX (FIX) in hemophilia B (HB), the latter being much less common. In fact, HB accounts for 15% of all diagnosed cases of hemophilia, with a prevalence of 1 per 40,000 live males.^1^, ^2^ Patients with hemophilia are characterized by a bleeding tendency, determined by factor levels, which severely affects their health and quality of life.^3^

The availability of new treatments, such as extended half‐life recombinant factors, has dramatically changed the clinical landscape for hemophilia patients.^4^ As a result, a life expectancy similar to that of the healthy population has been observed and, with it, the emergence of a greater number of age‐associated comorbidities, such as cardiovascular disease.^5^, ^6^, ^7^ Although it is true that hemophiliac patients have greater protection against thrombus formation due to factor deficiency, they are not exempt from cardiovascular disease such as obstructive artery disease due to atherosclerotic plaque formation or other risk factors.^8^ In fact, the group of Miesbach et al. observed that approximately 30% of patients over the age of 60 with moderate or severe hemophilia were diagnosed with cardiovascular disease, many of whom needed to undergo surgery.^9^

Cardiac surgery alone poses a high hemostatic risk, due to the need for total heparinization, surgical trauma, extracorporeal circulation (ECC), hypothermia, increased fibrinolysis, and cardiac arrest.^8^, ^9^ This type of procedure should be carried out with additional caution in hemophilia patients, due to their tendency to bleed. Current recommendations consider that the best approach is to individualize the treatment protocol and implement a multidisciplinary approach.^10^ However, the low number of hemophilia patients who have undergone cardiac surgery greatly limits the possibility of achieving consensus, especially in HB due to its very low incidence. The information currently available in the literature is very limited and mainly based on case reports and expert opinion.

Viscoelastic tests (VET) have also proven to be a very useful tool in the monitoring of patients during surgical procedures.^11^, ^12^ For example, the study by Vasquez et al. revealed that the use of rotational thromboelastometry (ROTEM®) to monitor patients undergoing cardiac surgery reduced the number of postoperative bleeds, the time from cardiopulmonary bypass discontinuation to skin suturing, and the length of intensive care unit stay.^13^

In general, HB patients who undergo surgery need to maintain hemostasis with recombinant FIX. The World Federation of Hemophilia (WFH) guidelines recommend FIX activity levels of 60–80 IU/dL prior to major surgery and continuous FIX replacement during the 14‐day postoperative period (40–60 IU/dL in the first 3 days, 30–50 IU/dL on days 4 to 6, and 20–40 IU/dL from days 7 to 14).^14^ In this regard, extended half‐life (EHL) FIX, such as albutrepenonacog alfa (rIX‐FP), has greatly facilitated the task, since it does not need to be administered as frequently as standard half‐life FIX.^15^ rIX‐FP is an rFIX genetically fused to human albumin, which allows for extended dosing intervals of 7–21 days and has shown good tolerability and very low bleeding rates.^16^ Recent studies reported the surgical experiences of the PROLONG‐9FP clinical program, revealing that rIX‐FP is safe and effective in the control of surgical and perioperative hemostasis. Overall, 30 surgeries (22 major) have been reported in 21 patients with severe HB with an overall success rate of 93.3%.^17^, ^18^, ^19^ However, no data have yet been published on cardiac surgery in a patient with HB receiving prophylactic treatment with rIX‐FP.

## 2 CASE REPORT

Our patient was a 48‐year‐old man with a diagnosis at 5 months of age of mild HB with a mutation in exon 6 of FIX and severe hemorrhagic phenotype; no history of inhibitor. He had a history of smoking, dyslipidemia, HIV (undetectable viral load) and treated HCV. The patient was receiving FIX on demand, although due to recurrent bleeding, he started tertiary prophylaxis with nonacog alfa (40 IU/kg, twice a week). The patient also had severe hemophilia‐associated arthropathy. Subsequently, he was switched to rIX‐FP (37.5 UI/kg per week) with the aim of improving his quality of life.

Two weeks after starting rIX‐FP, he attended the emergency department with abdominal pain in the right renal fossa, oppressive chest pain, and mild hypotension (104/76 mmHg). Laboratory tests were significant for elevated troponins at 23,161 ng/mL. The electrocardiogram showed ST segment elevation of 1 mm in II, III, avF and a decrease in aVL, and a diagnosis of acute coronary syndrome was given. On admission (D0) the patient presented a slight increase in activated partial thromboplastin time (aPTT) of 31.4 s (range, 20.6–31 s), coinciding with a trough FIX level of 27.3% (6 days since the last infusion). VET analysis with ROTEM (INTEM test: intrinsic pathway analysis) and Quantra® (QPlus Cartridge collecting intrinsic pathway analysis) showed a normal profile.

**FIGURE 1:**
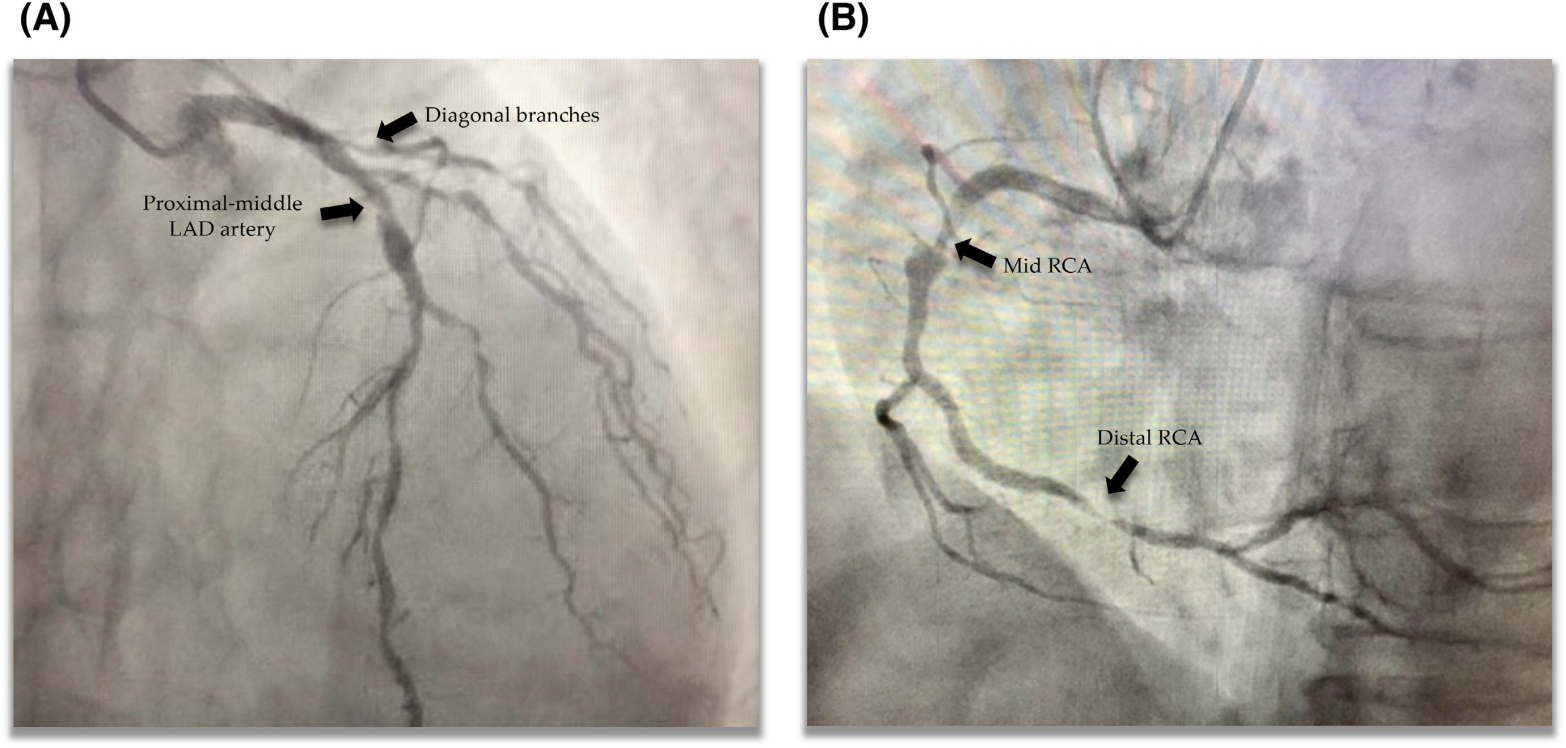
Cardiac catheterization findings. **(**A) Lesion in the left anterior descending (LAD) artery and diagonal branches. (B) Lesion in the mid right coronary artery (RCA) and distal RCA.

The patient started anticoagulation with enoxaparin at therapeutic doses (1 mg/kg every 12 h) and antiplatelet therapy with clopidogrel 75 mg every 24 h. An adverse thrombotic event related to prophylactic treatment was ruled out. FIX levels were closely monitored and maintained above 30% by rIX‐FP supplementation, avoiding very high levels due to concerns about thrombosis (Figure 1). Before catheterization, the patient received 12 IU/kg of rIX‐FP, and levels of 44.8% FIX were reached 19 h after infusion (D1). His ROTEM and Quantra profiles were normal, although a substantial reduction in clotting time were observed in Quantra (105 s) (Table 1).

**TABLE 1:** Viscoelastic test monitoring with respect to other clinical parameters during the perioperative process.

Time	FIX %	INTEM Test (ROTEM®)		QPlus cartridge (Quantra®)
		CT [100–240 s]	CFT [30–110 s]	CT [114–164 s]
D0: Admission	27.3	—	—	164
D1: 19 h after 12 IU/Kg of rIX‐FP	44.8	170	113	105
D8: 1 h after 25 IU/Kg of rIX‐FP	42.6	240	97	—
D8: 4 h after 25 IU/Kg of rIX‐FP	47.5	190	80	299
D14: pre‐ECC (1 h after 37.5 IU/Kg of rIX‐FP)	85.6	172	66	155
D14: post‐ECC (5 h after 37.5 IU/Kg of rIX‐FP)	74.5	217	92	147

Abbreviations: CFT, clot formation time; CT, clotting time; FIX, FIX level measured by one‐stage coagulation method; IU, international units; rIX‐FP, albutrepenonacog alfa; s, seconds.

The patient underwent catheterization via the right radial artery, revealing a coronary tree with severe, diffuse, calcified disease and well‐developed, diffusely diseased left anterior descending (LAD) artery with long severely calcified plaque in the proximal‐middle segment compromising the outflow of 2 well‐developed diagonal branches that also revealed proximal disease (Figure 1A). The circumflex artery had severe, diffuse disease throughout the proximal third extending into a marginal branch of borderline development. The dominant right coronary artery (RCA) showed a severe, apparently thrombotic lesion in the middle third, a severe lesion in the distal third, and severe and diffuse disease of the fine caliber posterior descending artery (Figure 1B). Systolic function was preserved.

**FIGURE 2:**
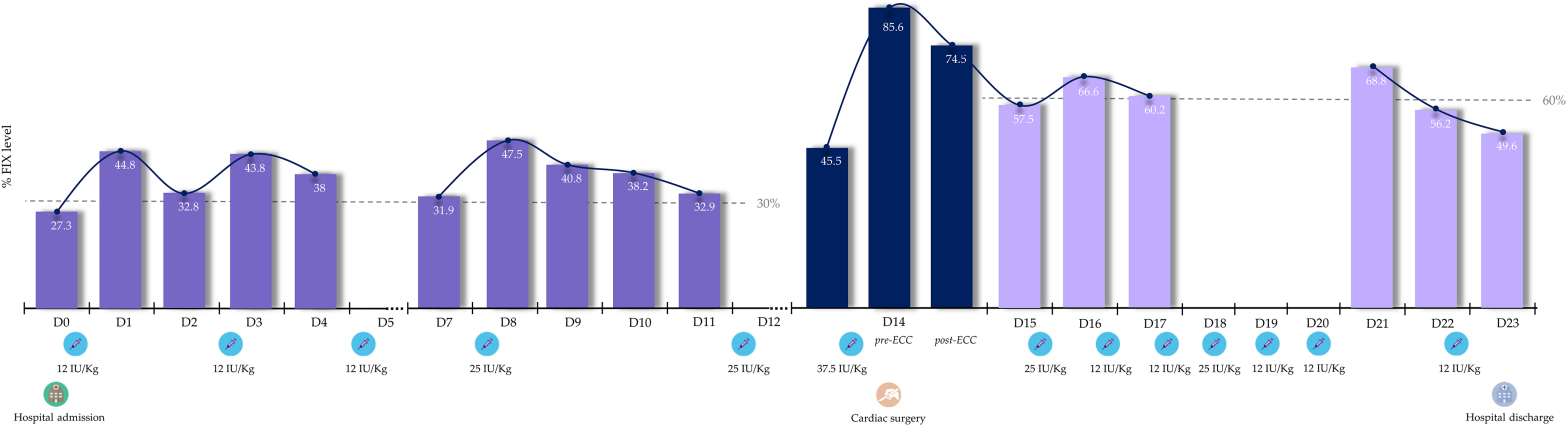
Monitoring of FIX levels. Monitoring of FIX levels from the day of admission until hospital discharge. On the Y‐axis, the percentage of FIX. On the X‐axis, days and rIX‐FP infusions. Infusions are placed to the left or right of the day, depending on whether these are administered before or after measuring FIX levels, respectively.

A coronary artery graft with cardiopulmonary bypass by cardiac surgery with ECC was proposed. Arterial/venous conduits used were (all of them in end‐to‐side anastomosis): saphenous vein graft to right coronary, saphenous vein graft (right) to first diagonal and left internal mammary artery to anterior descending coronary. Just before surgery (D14), the patient received 37.5 IU/kg of rIX‐FP, achieving FIX levels of 86.5%. Factor levels were maintained at 74.5% post‐ECC (Figure 2). D‐dimer values remained within the normal range and did not rise after rIX‐FP administration during the perioperative process (data not shown). Cardiac surgery was performed without complications and with excellent hemostasis, monitored by ROTEM and Quantra both pre‐ECC and post‐ECC, showing levels consistently within range (Table 1).

After surgery, the patient started treatment with antiplatelet therapy (acetylsalicylic acid 100 mg/day) and FIX levels remained at around 60% (D15 – D23). Eight infusions were administered in the 2‐week postoperative phase for a total of 147.5 IU/kg. After hospital discharge, the patient continued antiplatelet therapy. The patient's pharmacokinetics (PK) parameters showed a half‐life of rIX‐FP of 135.5 h, so we decided to prescribe 37.5 IU/kg once a week, with the aim of maintaining FIX levels above 30%. WAPPS‐Hemo V.5.4. was used to determine the individual PK profile of the patient at 4 FIX plasma levels (one‐stage coagulation method, using Actin FS activator and deficient plasma reagents of Siemens Healthcare, Erlangen, Germany) with no washout period: 30 min pre‐dose, and 3‐, 96‐ and 168‐hours post‐infusion. To date, the development of FIX inhibitors has not been observed.

## 3 DISCUSSION

In this article we report the case of a patient with a diagnosis of mild hemophilia B and severe hemorrhagic phenotype, who required surgery with ECC after being diagnosed with acute coronary syndrome.

The patient attended the emergency service due to an acute coronary syndrome just 2 weeks after changing treatment to rIX‐FP (37.5 IU/kg per week). Catheterization revealed a coronary tree with severe, diffuse, calcified disease, suggesting that this was not an adverse event associated with the prophylactic rIX‐FP. According to the literature, increasing numbers of hemophilia patients are requiring cardiac surgery because of longer life expectancies and accompanying age‐associated comorbidities such as cardiovascular disease.^5^, ^6^, ^7^ Further issues presented by the patient were a history of smoking, dyslipidemia, HIV, and HCV, all cardiovascular risk factors.^20^, ^21^, ^22^, ^23^

The indications for cardiac surgery in patients with hemophilia are the same as in patients without hemophilia, that is, there are no changes in surgical and anesthetic protocols.^24^ However, it should be noted that the procedure itself carries a high hemostatic risk and is particularly challenging in patients with hemophilia due to their tendency to bleed.^8^, ^9^ Heart surgery with ECC especially requires a more exhaustive control of hemostasis, since it involves heparinization of the patient at full doses to ensure blood circulation through the external circuit.^25^ Therefore, the main challenge during this type of procedure in patients with hemophilia is to decide the amount of anticoagulation, which will be directly influenced by the levels of deficient factor. Nevertheless, there are no well‐defined protocols on the management of these patients during this type of procedure, which gives relevance to this case. Indeed, reports of cardiac surgery in patients with hemophilia, especially hemophilia B, are very scarce, and are limited to case reports, small series, or expert opinions. Most recent reviews have focused on perioperative management but fail to address optimal strategies for the management of these patients during surgery.^26^, ^27^, ^28^

Cardiac surgery with and without ECC has been shown to be effective and safe. However, in expert hands, surgery without ECC has been shown to reduce morbidity and, in nonrandomized adjusted studies, mortality in high‐risk patients.^29^ Despite the great advantage of avoiding manipulation of the aorta during clamping in a patient with a high risk of bleeding, the higher experience in surgery with ECC of our center led us to perform the intervention using this protocol.

Most experts agree that cardiac surgery in patients with hemophilia requires a multidisciplinary and highly individualized approach for each patient, with the participation of hematologists, anesthesiologists, surgeons, intensivists, and laboratory specialists.^26^, ^28^ It should be noted that this patient's surgery was scheduled for a Monday, because, as recommended by some authors, performing surgery at the beginning of the week helps to ensure the availability of all personnel and thus more satisfactory patient outcomes.^30^, ^31^, ^32^

With regard to therapeutic interventions during the surgical process, we found that optimization of factor levels before, during and after surgery was essential to obtain results similar to those obtained in non‐hemophiliac patients.^33^ In the run‐up to surgery, we decided to maintain FIX levels above 30%, while avoiding very high peaks, due to concerns about the risk of thrombosis. On the day of surgery, a higher dose was administered, following the recommendations of the WFH guidelines, to achieve FIX levels close to 80%.^14^ The administration of a single preoperative bolus of rIX‐FP was sufficient to maintain excellent hemostasis, and surgery could be performed with neither hemorrhagic nor thrombotic complications, demonstrating that a single bolus can maintain high FIX levels during the procedure.^17^ Moreover, the patient did not require an additional dose until 24 h after the intervention. The study by Curtin et al. described the experience of the PROLONG‐9FP surgical substudy and concluded that treatment with rIX‐FP offers patients effective protection against perioperative bleeding. This study reported experience from 21 major interventions, although none of them involved cardiac surgery. In this report we present the first case of a hemophilia B patient receiving rIX‐FP treatment for cardiac surgery with ECC, demonstrating that rIX‐FP also offers protection from perioperative bleeding to patients in this type of intervention. During the 2 weeks after surgery, the patient required low FIX doses (147.5 IU/kg of rIX‐FP), which was considerably lower than the median dose of 221.7 (0–444.07) IU/kg reported by Curtin et al.^17^ Nevertheless, it should be mentioned that the patient was also maintained on antiplatelet therapy after surgery. These results further support the evidence that EHL products, such as rIX‐FP, offer a promising alternative to standard half‐life (SHL) drugs in patients undergoing surgery, as they reduce the number of infusions, doses, and costs, especially in the case of EHL FIX.^34^

VETs with ROTEM and Quantra were especially useful to evaluate the global hemostatic status during the perioperative process, allowing us to analyze components such as platelet and fibrinogen levels, beyond the effect of FIX levels. In addition, VET proved very useful for monitoring the effect of heparin during surgery. These tools have been widely used in the surgical setting, especially for the differential diagnosis of post‐surgical bleeding in disorders such as hypofibrinogenemia or thrombocytopenia.^35^, ^36^, ^37^ The values measured by both techniques remained within the normal range, with FIX levels always above 30%. Previous studies have reported normal thromboelastography values in patients with factor levels below 10%.^38^, ^39^ There was good agreement between the results of Quantra and ROTEM, which demonstrates that both techniques are potentially interesting in this setting. Viscoelastic techniques are thus positioned as a plausible alternative to conventional techniques such as activated partial thromboplastin time (aPTT) and specific assays for FVIII and FIX. The use of aPTT during cardiopulmonary bypass is not feasible, as heparin concentrations of >1 U/mL will disrupt aPTT measurements, while FVIII or FIX assays cannot be performed quickly enough during cardiac surgery.^26^

In summary and to the best of our knowledge, this is the first report of cardiac surgery with ECC in a patient with hemophilia B receiving treatment with rIX‐FP. Based on the success of this patient's surgery and the suggestions of other authors regarding cardiac surgery in patients with hemophilia, we consider that there are a series of considerations that are essential for the management of this type of patient in these situations: (1) surgery should be scheduled under the supervision of a multidisciplinary team with experience in this field, (2) treatment should be individualized following current WHO recommendations, (3) treatment should be closely monitored to ensure good hemostatic protection during and after surgery, (4) viscoelastic testing during the perioperative process is very useful to assess the patient's overall hemostatic status, and (5) treatment with EHL FIX, such as rIX‐FP, in patients with HB is safe and effective in this type of surgery. In fact, a single administration of rIX‐FP pre‐intervention was successful in our case in maintaining elevated FIX levels and preventing bleeding during surgery and even in the 24 h after the intervention.

## 4 CONCLUSION

In a patient with hemophilia B, cardiac surgery with extracorporeal circulation was performed safely without hemorrhagic or thrombotic complications after a single preoperative dose of rIX‐FP. Viscoelastic tests proved to be very useful in monitoring the patient during surgery.

## AUTHOR CONTRIBUTIONS

**Francisco José López‐Jaime:** Conceptualization; funding acquisition; project administration; supervision; writing – review and editing. **Ihosvany Fernández‐Bello:** Investigation; writing – review and editing. **Eva Calavia‐Aranda:** Data curation; writing – review and editing. **Begoña Reina Monsó:** Investigation; supervision; writing – review and editing. **Adrián Montaño:** Conceptualization; data curation; funding acquisition; writing – original draft; writing – review and editing.

## CONFLICT OF INTEREST STATEMENT

Francisco J. López Jaime has participated as consultant or speaker for Amgen, Bayer, CSL Behring, Leo Pharma, Novartis, Novo Nordisk, Pfizer, Roche, Sobi and Takeda. The rest of the authors declare that they have no interests that could be perceived as a conflict or bias.

## CONSENT

Written, informed consent was obtained from the patient to publish this report in accordance with the journal's patient consent policy.

## Data Availability

Data available on request from the authors.
